# Association Between Renal Dysfunction and Cerebral Small Vessel Disease: A Prospective Cohort Study From the UK Biobank

**DOI:** 10.1002/cns.70802

**Published:** 2026-02-24

**Authors:** Xiaowei Sun, Jiawen Chen, Yang Gao, Yingjie Chen, Yan Li, Rui Pang, Ling Cai, Zhangsheng Yu, Dan Huang, Peiying Li

**Affiliations:** ^1^ Clinical Research Center, Renji Hospital Shanghai Jiao Tong University School of Medicine Shanghai China; ^2^ Department of Anesthesiology, Renji Hospital Shanghai Jiao Tong University School of Medicine Shanghai China; ^3^ Institute of Clinical Medicine Shanghai Jiao Tong University School of Medicine Shanghai China

**Keywords:** cerebral small vessel disease, fractional anisotropy, mean diffusivity, renal dysfunction, white matter injury

## Abstract

**Aims:**

This study aims to test the association between multidimensional renal dysfunction biomarkers and cerebral small vessel disease (CSVD) risk using data from the UK Biobank.

**Methods:**

The present study encompasses two cohorts. The whole cohort consisted of 43,314 adults without neurological diseases at baseline, who underwent brain magnetic resonance imaging (MRI) during the follow‐up period. CSVD imaging markers, including white matter hyperintensity (WMH) volume, mean diffusivity (MD), and fractional anisotropy (FA), were extracted. The sub‐cohort consisted of 9786 adults randomly selected from the whole cohort. CSVD MRI features, including the presence of lacunes, white matter hyperintensities (WMHs), enlarged perivascular spaces (EPVS), and cerebral microbleeds (CMBs), as well as CSVD burden score, were assessed.

**Results:**

In the whole cohort, renal dysfunction as reflected by abnormalities in estimated glomerular filtration rates and blood urea nitrogen was associated with increased WMH and MD values and decreased FA values, compared to the healthy group. We observed consistent findings in the sub‐cohort: multidimensional renal dysfunction was associated with increased risk of lacunes, WMHs, EPVS, and CMBs, as well as greater severity of CSVD burden.

**Conclusions:**

Our large‐scale epidemiological study provides evidence that multidimensional renal dysfunction biomarkers are independently associated with CSVD risk in adults.

## Graphical abstract

In the whole cohort, renal dysfunction was associated with increased WMH and MD values, and decreased FA values, which suggests a higher risk of CSVD. Consistent findings were observed in the sub‐cohort: renal dysfunction was associated with increased risk of lacunes, WMHs, EPVS, and CMBs, as well as greater severity of CSVD burden.

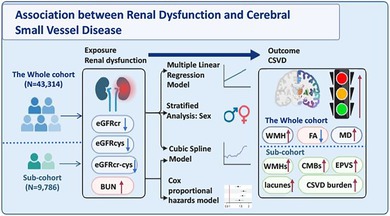

## 1 Introduction

Cerebral small vessel disease (CSVD) is a major public health concern worldwide, accounting for 25% of ischaemic stroke and 45% of dementia cases [1, 2]. It is associated with various neurological diseases and related disabilities [3, 4, 5]. Diverse features of CSVD are visible on multimodal magnetic resonance imaging (MRI). White matter hyperintensities (WMHs), lacunes, cerebral microbleeds (CMBs), and enlarged perivascular spaces (EPVS) are typical imaging markers of CSVD. Emerging CSVD features such as diffusion tensor imaging (DTI) metrics‐ mean diffusivity (MD) and fractional anisotropy (FA) can better detect associations with clinical deficits in CSVD than conventional markers [6, 7]. Advances in neuroimaging techniques have led to increased diagnosis of CSVD in the older population [8, 9]. Despite its high prevalence and substantial clinical impact, CSVD pathogenesis remains poorly understood. In the absence of effective treatments, identifying risk factors and developing preventive strategies are critical for reducing CSVD‐related morbidity.

The association between renal dysfunction and CSVD was recently proposed. Renal dysfunction, such as chronic kidney disease (CKD), is a global health burden that affects more than 10% of the general population [10] and nearly 40% of people aged over 60 years [11]. Renal function can be assessed using serum biomarkers, with the creatinine‐based estimated glomerular filtration rate (eGFR_Cr_) being the most widely used. Cystatin C is an alternative biomarker of renal function (eGFR_Cys_) and can be combined with creatinine (eGFR_Cr‐Cys_) [12]. Renal dysfunction can lead to an increased production of circulating pro‐inflammatory cytokines, which are independent risk factors for incident cardiovascular disease [13]. Increased inflammation has been associated with white matter damage in various neurological diseases, including CSVD [14, 15, 16, 17]. The association between renal function biomarkers and stroke, cardiovascular disease, cognitive decline, and dementia has been extensively documented [18, 19, 20, 21]. Studies exploring the association between renal function and CSVD have mainly focused on patients with specific diseases, showing that low eGFR is linked to higher CSVD burden in primary intracerebral hemorrhage and hypertension, while cystatin C levels are positively associated with white matter abnormalities in patients with cognitive impairment [22, 23, 24]. Currently, the association between multidimensional renal dysfunction biomarkers and CSVD, based on large‐scale epidemiological evidence, remains unclear. Only several cross‐sectional studies with limited sample sizes revealed that cystatin C and eGFR are risk factors for conventional CSVD imaging features, such as CSVD burden, WMHs, lacunas, and perivascular spaces [25, 26, 27].

Therefore, using a large sample size and comprehensive data on individual characteristics, renal function, and brain imaging from the UK Biobank, we aim to test the association of multidimensional renal dysfunction (eGFR_Cr_, eGFR_Cys_, eGFR_Cr‐Cys_, and blood urea nitrogen [BUN]) with the risk of CSVD in adults.

## 2 Methods

### 2.1 Study Design and Participants

**FIGURE 1:**
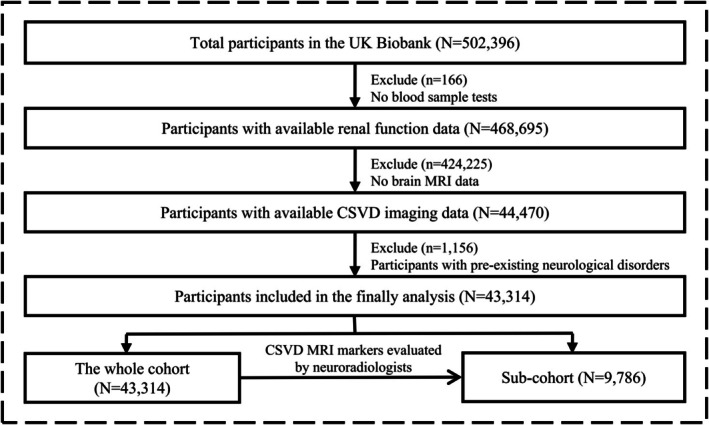
Study flow diagram for participant inclusion.

The UK Biobank is a large‐scale, population‐based cohort study comprising 502,536 participants aged 37–73 years who were recruited between 2006 and 2010. All participants signed informed consent forms, which were approved by the North West Multi‐centre Research Ethics Committee. The present study was carried out utilizing the UK Biobank Resource under application Number 100014. Data on demographic characteristics, physical and functional measures, and genetic and biological assessments were collected across the United Kingdom. The UK Biobank initiated an imaging study in 2014. For the main analysis, we included participants without chronic neurological diseases and dementia at baseline (Table S1) and who underwent brain MRI during the follow‐up period. The detailed study flow and participant inclusion criteria are summarized in Figure 1.

### 2.2 Multidimensional Renal Dysfunction Assessment

The eGFR_Cr_, eGFR_Cys_, and eGFR_Cr‐Cys_ levels were calculated using the Chronic Kidney Disease Epidemiology Collaboration (CKD‐EPI) equation. According to the Kidney Disease: Improving Global Outcomes (KDIGO) [28], an eGFR cutoff of 90 mL/min/1.73 m^2^ was used, with values below this threshold indicating mild kidney damage. In addition to glomerular filtration function, BUN was included as it may have independent prognostic value for assessing renal tubule reabsorption. A BUN cutoff of 20 mg/dL has been established, where BUN > 20 mg/dL indicates impaired renal excretion function or a protein metabolism disorder [29]. Detailed renal function calculations are described in Appendix S1.

### 2.3 Assessment of CSVD Using MRI

We used the multimodal MRI data released by the UK Biobank in February 2023. Data from 43,314 participants collected between 2014 and 2015 were analyzed in the whole cohort. CSVD MRI features were assessed using the conventional marker WMH volume and DTI metrics: white matter FA and MD in 48 white matter tracts. WMH volumes were log‐transformed to approximate a normal distribution. We conducted principal component analysis of various white matter tracts to obtain a comprehensive assessment of white matter FA and MD. The first principal components of white matter FA and MD were used in the analyses.

To accurately elucidate the association between multidimensional renal function biomarkers and risk of CSVD, we conducted analyses in the sub‐cohort. The sub‐cohort consisted of 9786 adults randomly stratified by age and sex from the whole cohort. Eligibility criteria included the availability of all three MRI sequences (T1‐weighted imaging, fluid‐attenuated inversion recovery, and susceptibility‐weighted imaging), complete baseline clinical information, and no history of neurological diseases. CSVD MRI features, including the presence of lacunes, WMHs, EPVS, and CMBs, were assessed by two independent neuroradiologists with over 10 years' medical imaging experience based on the Standards for Reporting Vascular Changes on Neuroimaging (STRIVE) criteria [9]. Any discrepancies were resolved through discussion. The total CSVD burden was quantified using a validated composite score (range 0–4), with one point assigned for the presence of each of the following markers: ≥ 1 lacune; deep WMH Fazekas score ≥ 2 or periventricular WMH Fazekas score of 3; moderate to severe EPVS in the basal ganglia; ≥ 1 deep CMB [30]. Higher composite scores indicated greater overall CSVD burden.

### 2.4 Mendelian Randomization (MR) Study Design

Two‐sample MR was used to assess the effects of multidimensional renal function (eGFR_Cr_ and eGFR_Cys_) biomarkers on CSVD risk. Genetic variants associated with eGFR_Cr_ and eGFR_Cys_ were selected as instrumental variables for the MR analysis, based on a previous study [31].

Renal function data were sourced from the UK Biobank and CKDGen Consortium (www.genepi‐regensburg.de/ckd), encompassing 1,201,909 individuals and 13,633,840 single‐nucleotide polymorphisms (SNPs) [32]. Data on CSVD MRI features were derived from the UK Biobank, WMH stroke study, and CHARGE consortium (www.kp4cd.org/dataset_downloads/stroke), including 42,310 individuals and 9,733,965 SNPs [7].

### 2.5 Statistical Analyses

Descriptive statistics were used to summarize demographics, lifestyle, multidimensional renal function biomarkers, and CSVD MRI features. A cubic spline model was used to examine potential nonlinear associations between multidimensional renal function biomarkers (eGFR_Cr_, eGFR_Cys_, eGFR_Cr‐Cys_, and BUN) and CSVD (WMH volume, white matter FA and MD in the whole cohort; the presence of lacunes, WMHs, EPVS, CMBs, and total CSVD burden in the sub‐cohort). Subsequently, renal function biomarkers were divided into low (eGFR < 90 mL/min/1.73 m^2^; BUN < 20 mg/dL) and high (eGFR ≥ 90 mL/min/1.73 m^2^; BUN ≥ 20 mg/dL) groups based on the literature.

First, to investigate the association between multidimensional renal function biomarkers and CSVD risk, we conducted analyses in the whole cohort, in which CSVD risk was reflected by WMH volume, white matter FA, and white matter MD. Multiple linear regression models were used to examine the associations of eGFR_Cr_, eGFR_Cys_, eGFR_Cr‐Cys_, and BUN with WMH volume, white matter FA, and white matter MD. We included an interaction term between each renal function biomarker and sex in the regression model to explore the possible sex‐specific effects, with results presented separately for sex. We used a false discovery rate (FDR) correction to deal with multiple comparisons and reported the FDR‐corrected *p*‐values.

Second, we conducted analyses in the sub‐cohort to accurately elucidate the association between multidimensional renal function biomarkers and CSVD. In the sub‐cohort, CSVD was defined by the presence of lacunes, WMHs, EPVS, and CMBs. The total CSVD burden score was then used to classify disease severity into low (≤ 1) and high (> 1) categories. Using Cox proportional hazards regression models, with the time blood samples were collected as the start of follow‐up, we further investigate the association of eGFR_Cr_, eGFR_Cys_, eGFR_Cr‐Cys_, and BUN with lacunes, WMHs, EPVS, and CMBs, as well as the total CSVD burden.

Next, we applied a two‐sample MR approach to investigate the potential causal associations of eGFR_Cr_ and eGFR_Cys_ with WMH volume. SNPs satisfying genome‐wide significance (*p* < 5 × 10^−8^) with F‐statistics > 600 (eGFR_Cr_) or > 3000 (eGFR_Cys_) were used for MR analyses. Cochran's *Q* test was used to identify heterogeneity. The MR‐Egger intercept test was used to evaluate horizontal pleiotropy. The primary MR analysis relied on the inverse variance‐weighted (IVW) method. MR‐Egger and weighted median (WME) methods were used for sensitivity analyses to test the robustness of the results. Additionally, we applied the MR‐PRESSO method to identify potential outliers and used the leave‐one‐out analysis to assess the impact of specific SNPs. The effect estimates of genetically predicted eGFR_Cr_ and eGFR_Cys_ on WMH volume were presented as ORs or β‐coefficients with 95% CIs.

Additionally, several exploratory analyses were performed to evaluate the association between multidimensional renal function biomarkers and CSVD across subgroups stratified by diet‐related inflammation (measured by dietary inflammatory index, DII) and chronic comorbidities, including hypertension, diabetes, and dyslipidaemia in the whole cohort. A total of 29 foods and nutrients available in the UK Biobank were included to calculate the DII score (Table S2), as described elsewhere [33]. To assess the potential effect modification, interaction terms (renal function markers × DII score or hypertension or diabetes, or dyslipidemia) were incorporated into the regression models. We examined the joint associations of renal function markers, DII categories [defined as anti‐inflammatory diet group (DII score ≤ 0) and pro‐inflammatory diet group (DII score > 0)], and chronic comorbidities categories with CSVD using multiple regression models.

The present study followed the Strengthening the Reporting of Observational Studies in Epidemiology (STROBE) reporting guideline for cohort studies. We conducted the analyses using SAS version 9.4 and R version 4.3.1. Two‐sided *p*‐values < 0.05 were considered statistically significant.

## 3 Results

### 3.1 Characteristics of Participants

Characteristics of the participants in the whole cohort and sub‐cohort are presented in Table 1. In the whole cohort, 43,314 participants (mean [SD] age, 54.99 [7.54] years; 22,928 females [52.93%]; 20,386 males [47.07%]) with renal function and CSVD assessment were included, with a median follow‐up of 9.48 years (IQR, 7.92–10.64) (Table 1). The mean (SD) concentrations of eGFR_Cr_, eGFR_Cys_, eGFR_Cr‐Cys_, and BUN were 91.75 (11.46), 90.83 (13.94), 95.12 (12.41) mL/min/1.73 m^2^, and 14.87 (3.45) mg/dL, respectively. Detailed data are presented in Table S3. Characteristics of participants in the sub‐cohort (*N* = 9786) were compatible with those without the sub‐cohort (*N* = 33,528) in general, and those results were presented in Table S4.

**TABLE 1:** Characteristics of the included participants.

Characteristics	The whole cohort	Sub‐cohort
	Mean ± SD or *N* (%)	
Age	54.99 (7.54)	54.70 (7.48)
**Sex**		
Male	20,386 (47.07)	4626 (47.27)
Female	22,928 (52.93)	5160 (52.73)
**BMI, kg/m** ^**2**^	26.56 (4.21)	26.40 (4.10)
**Race**		
White British	39,460 (91.13)	8937 (91.34)
Other	3843 (8.87)	847 (8.66)
**Tobacco smoking**		
Never smoked	26,351 (60.84)	6189 (63.26)
Previous	14,222 (32.83)	3128 (31.97)
Current	2648 (6.11)	448 (4.58)
Unknown	93 (0.21)	19 (0.19)
**Alcohol drinking**		
Never drank	1050 (2.42)	245 (2.50)
Previous	891 (2.06)	154 (1.57)
Current	41,349 (95.46)	9383 (95.90)
Unknown	24 (0.06)	2 (0.02)
**Household income range, £**		
< 18,000	4493 (10.37)	891 (9.24)
18,000–30,999	8591 (19.83)	1925 (19.96)
31,000–51,999	11,800 (27.24)	2687 (27.86)
52,000–100,000	11,127 (25.69)	2651 (27.49)
> 100,000	3056 (7.06)	713 (7.39)
Unknown	4247 (9.81)	776 (8.05)
**Hypertension**		
Yes	9462 (25.50)	1791 (21.78)
No	27,642 (74.50)	6433 (78.22)
**Diabetes**		
Yes	2011 (5.42)	345 (4.20)
No	35,093 (94.58)	7879 (95.80)
**Dyslipidemia**		
Yes	4906 (13.22)	868 (10.55)
No	32,198 (86.78)	7356 (89.45)
**Renal function**		
eGFR_Cr_	91.75 (11.46)	91.85 (11.34)
eGFR_Cys_	90.83 (13.94)	91.33 (13.62)
eGFR_Cr‐Cys_	95.12 (12.41)	95.44 (12.15)
BUN	14.87 (3.45)	14.83 (3.40)

Abbreviations: BMI, body mass index; BUN, blood urea nitrogen; eGFR_Cr_, estimated glomerular filtration rate based on creatinine; eGFR_Cys_, estimated glomerular filtration rate based on cystatin C; eGFR_Cr‐Cys_, estimated glomerular filtration rate based on creatinine and cystatin C.

### 3.2 Association Between Multidimensional Renal Dysfunction Biomarkers and CSVD

**FIGURE 2:**
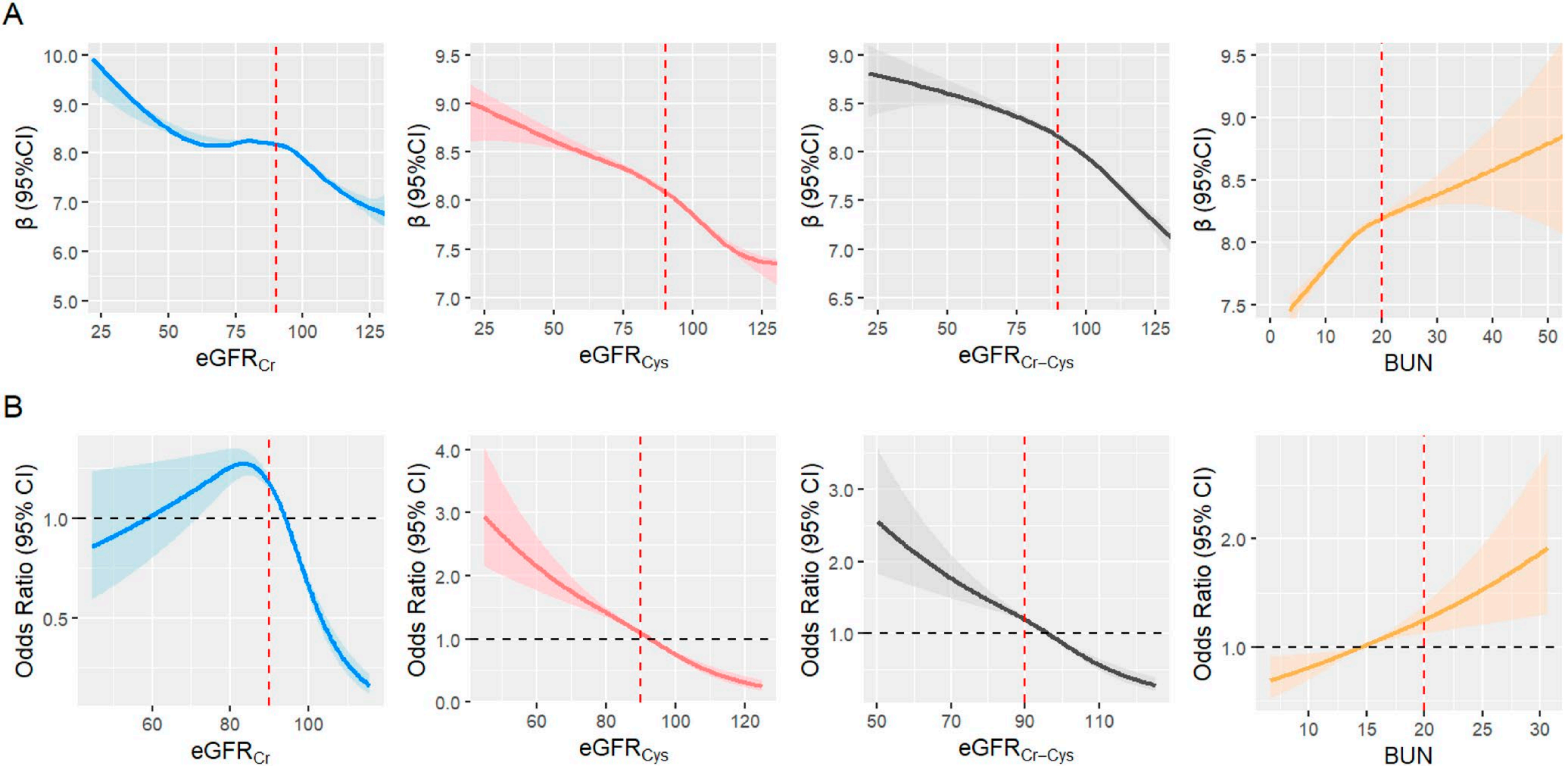
Restricted Cubic spline model of renal function and CSVD. (A) Restricted cubic spline model of renal function and white matter hyperintensity volumes in the whole cohort. (B) Restricted cubic spline model of renal function and CSVD burden in sub‐cohort. (1) BUN, blood urea nitrogen; EGFR_Cr_, estimated glomerular filtration rate based on creatinine; eGFR_Cr‐Cys_, estimated glomerular filtration rate based on creatinine and cystatin C; eGFR_Cys_, estimated glomerular filtration rate based on cystatin C; WMH, white matter hyperintensities. (2) The blue line represents eGFR_Cr_, the red line represents eGFR_Cys_, the black line represents eGFR_Cr‐Cys_, and the yellow line represents BUN. The dotted line represents health status, eGFR_Cr_ ≥ 90, eGFR_Cys_ ≥ 90, eGFR_Cr‐Cys_ ≥ 90, and BUN < 20.

The restricted cubic spline models showed nonlinear associations between multidimensional renal function biomarkers and CSVD (WMH volume, white matter FA and MD in the whole cohort; lacunes, WMHs, EPVS, CMBs, and total CSVD burden in the sub‐cohort) in several models (Figure 2; Figures S1 and S2). Then, renal function biomarkers were divided into renal dysfunction (eGFR < 90 mL/min/1.73 m^2^; BUN > 20 mg/dL) and healthy (eGFR ≥ 90 mL/min/1.73 m^2^; BUN < 20 mg/dL) groups.

First, we conducted analyses using data from the whole cohort. In the multiple linear regression models, after adjusting for household income, BMI, race, tobacco smoking, alcohol drinking, and brain volume, renal dysfunction was associated with increased WMH volumes as compared with the healthy groups; the estimated β coefficients (95% CI) for eGFR_Cr_, eGFR_Cys_, eGFR_Cr‐Cys_, and BUN were 0.177 (0.157, 0.196), 0.348 (0.328, 0.367), 0.297 (0.276, 0.318), and 0.031 (−0.003, 0.065), respectively. We then examined the association between multidimensional renal function biomarkers and the first principal components of DTI white matter FA and MD. Renal dysfunction, as defined by eGFR_Cr_, eGFR_Cys_, eGFR_Cr‐Cys_, and BUN levels, was associated with decreased white matter FA and increased MD values. Next, we performed a sex‐stratified analysis and found similar associations in males and females, except that the associations of BUN levels with WMH volumes and white matter MD were more prominent in females (Table 2). When further adjusting for sleep disorder and medication history in the regression model, similar results were observed (Table S5).

**TABLE 2:** The association between renal function and CSVD imaging markers in the whole cohort.

Renal function	All			Male			Female		
	*β*	95% CI	*p* (*p* for FDR)	*β*	95% CI	*p* (*p* for FDR)	*β*	95% CI	*p* (*p* for FDR)
**WMH**									
eGFR_Cr_ ^a^	Ref		Ref	Ref					
	0.177	0.157, 0.196	< 0.001	0.188	0.160, 0.217	< 0.001	0.158	0.131, 0.184	< 0.001
eGFR_Cys_ ^a^	Ref		Ref	Ref					
	0.348	0.328, 0.367	< 0.001	0.286	0.257, 0.314	< 0.001	0.376	0.349, 0.403	< 0.001
eGFR_Cr‐Cys_ ^a^	Ref		Ref	Ref					
	0.297	0.276, 0.318	< 0.001	0.262	0.232, 0.291	< 0.001	0.314	0.285, 0.342	< 0.001
BUN^b^	Ref		Ref	Ref					
	0.031	−0.003, 0.065	0.107	−0.010	−0.054, 0.034	0.845	0.094	0.040, 0.147	< 0.001
**FA**									
eGFR_Cr_ ^a^	Ref		Ref	Ref					
	−0.527	−0.615, −0.439	< 0.001	−0.568	−0.698, −0.438	< 0.001	−0.490	−0.610, −0.371	< 0.001
eGFR_Cys_ ^a^	Ref		Ref	Ref					
	−0.922	−1.009, −0.834	< 0.001	−0.832	−0.960, −0.704	< 0.001	−1.023	−1.144, −0.901	< 0.001
eGFR_Cr‐Cys_ ^a^	Ref		Ref	Ref					
	−0.869	−0.961, −0.776	< 0.001	−0.813	−0.947, −0.679	< 0.001	−0.925	−1.053, −0.797	< 0.001
BUN^b^	Ref		Ref	Ref					
	−0.377	−0.536, −0.218	< 0.001	−0.317	−0.524, −0.110	< 0.001	−0.465	−0.716, −0.215	0.004
**MD**									
eGFR_Cr_ ^a^	Ref		Ref	Ref					
	0.671	0.580, 0.761	< 0.001	0.733	0.600, 0.866	< 0.001	0.578	0.456, 0.699	< 0.001
eGFR_Cys_ ^a^	Ref		Ref	Ref					
	1.298	1.208, 1.388	< 0.001	1.140	1.010, 1.272	< 0.001	1.289	1.165, 1.413	< 0.001
eGFR_Cr‐Cys_ ^a^	Ref		Ref	Ref					
	1.176	1.081, 1.271	< 0.001	1.119	0.982, 1.256	< 0.001	1.143	1.012, 1.274	< 0.001
BUN^b^	Ref		Ref	Ref					
	0.201	0.042, 0.360	0.018	0.146	−0.061, 0.354	0.236	0.284	0.033, 0.535	0.045

*Note:* Defined by renal function: ref: eGFR_Cr_ ≥ 90, eGFR_Cys_ ≥ 90, eGFR_Cr‐Cys_ ≥ 90, and BUN < 20.

Abbreviations: BUN, blood urea nitrogen; eGFR_Cr_, estimated glomerular filtration rate based on creatinine; eGFR_Cys_, estimated glomerular filtration rate based on cystatin C; eGFR_Cr‐Cys_, estimated glomerular filtration rate based on creatinine and cystatin C; FA, fractional anisotropy; MD, mean diffusivity; WMH, white matter hyperintensities.

^a^ Models adjusted for household income, BMI, race, tobacco smoking, alcohol drinking, and brain volume.

^b^ Models adjusted for age, sex, household income, BMI, race, tobacco smoking, alcohol drinking, and brain volume.

**FIGURE 3:**
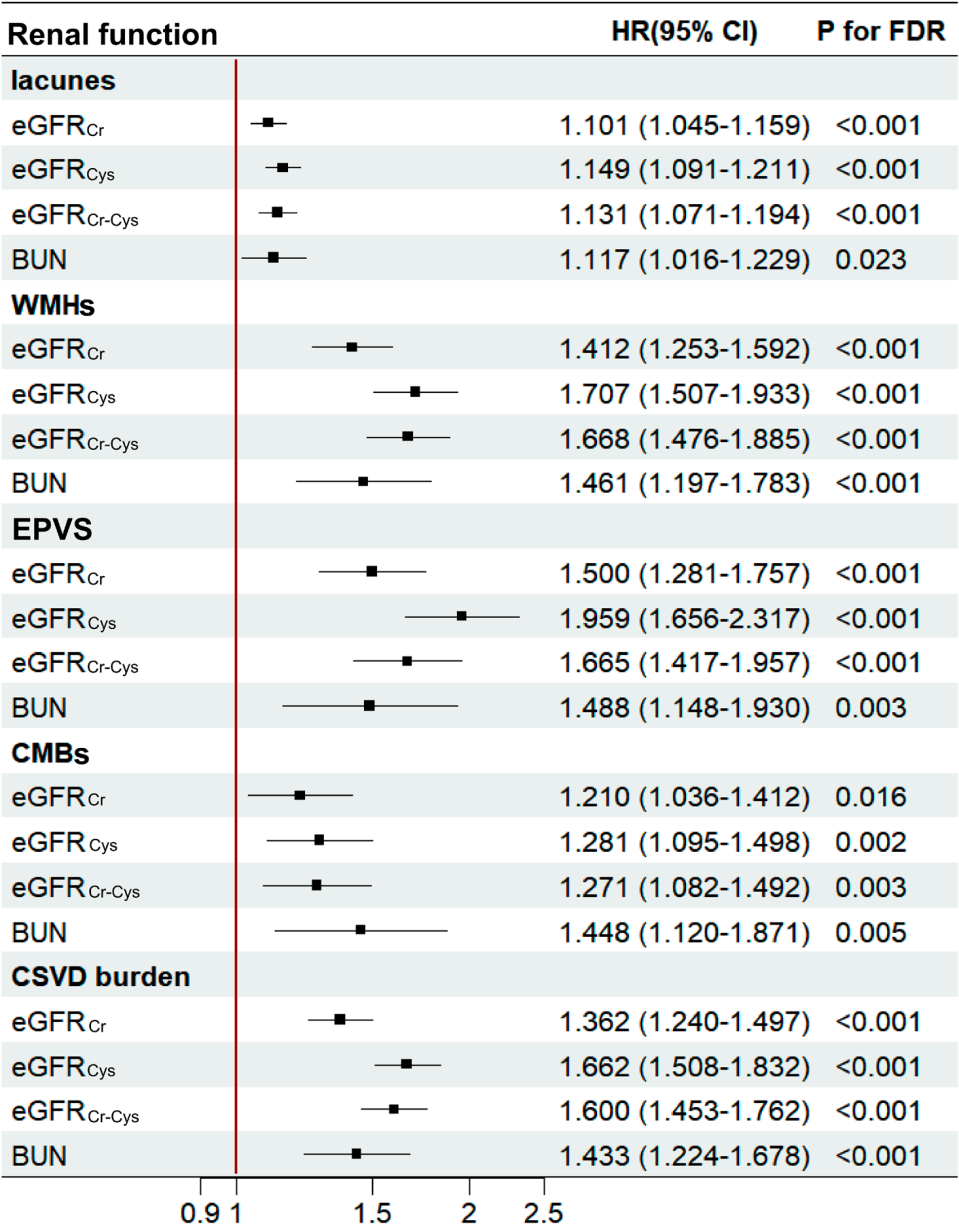
The association between renal function and CSVD imaging markers in the sub‐cohort. (1) BUN, blood urea nitrogen; CMBs, cerebral microbleeds; CSVD, cerebral small vessel disease; EGFR_Cr_, estimated glomerular filtration rate based on creatinine; eGFR_Cr‐Cys_, estimated glomerular filtration rate based on creatinine and cystatin C; eGFR_Cys_, estimated glomerular filtration rate based on cystatin C; EPVS, enlarged perivascular space; WMHs, white matter hyperintensities. (2) eGFR_Cr_, eGFR_Cys_, and eGFR_Cr‐Cys_ models adjusted for household income, BMI, race, tobacco smoking, alcohol drinking, and brain volume. BUN models adjusted for age, sex, household income, BMI, race, tobacco smoking, alcohol drinking, and brain volume. (3) Defined by renal function: Ref. EGFR_Cr_ ≥ 90, eGFR_Cys_ ≥ 90, eGFR_Cr‐Cys_ ≥ 90, and BUN < 20.

Second, the analyses were performed using data from the sub‐cohort. Compared to the healthy groups, renal dysfunction, as reflected by eGFR_Cr_, eGFR_Cys_, eGFR_Cr‐Cys_, and BUN, had a higher probability of belonging to the high CSVD burden groups (Table S6). In Cox proportional hazards regression models, the HR (95% CI) for eGFR_Cr_, eGFR_Cys_, eGFR_Cr‐Cys_, and BUN were 1.362 (1.240, 1.497), 1.662 (1.508, 1.832), 1.600 (1.453, 1.762), and 1.433 (1.224, 1.678), respectively (Figure 3). As for the specific CSVD imaging markers, renal dysfunction, as defined by eGFR_Cr_, eGFR_Cys_, eGFR_Cr‐Cys_, and BUN levels, was associated with increased risk of lacunes, WMHs, EPVS, and CMBs (Figure 3). In terms of hazard ratios (HRs), the strength of association varied across components: Lacunes (HR range: 1.101–1.149), WMH (HR range: 1.412–1.707), EPVS (HR range: 1.488–1.959), and CMB (HR range: 1.210–1.448).

### 3.3 Two‐Sample MR Analyses

Using two‐sample MR analyses, we further demonstrated potential causal associations between multidimensional renal function biomarkers and CSVD. The selected SNPs associated with eGFR_Cr_ and eGFR_Cys_ explained 1.62% and 5.32% of phenotypic variance, respectively, supporting their adequacy as instrumental variables. Cochran's *Q* test revealed no heterogeneity, and the MR‐Egger intercept test indicated no horizontal pleiotropy. The random‐effects IVW models showed that genetically predicted increases in eGFR_Cr_ and eGFR_Cys_ were associated with lower WMH volumes (OR_eGFRCr_ = 0.713, 95% CI: 0.604–0.842; OR_eGFRCys_ = 0.879, 95% CI: 0.848–0.910). Similar associations were observed in the MR‐Egger and WME models (Figures S3 and S4). Additionally, the MR‐PRESSO and leave‐one‐out analyses identified no outliers, supporting the robustness of our findings.

### 3.4 Stratification Analyses

We further performed stratification analyses to assess the robustness of the association across subgroups (Tables S6–S9). The joint association of multidimensional renal function biomarkers and DII categories with CSVD was examined using multiple regression models. Compared to the healthy groups, individuals in the renal dysfunction groups had increased WMH volumes in both strata of the DII categories, with the positive associations of eGFR_Cr_ and eGFR_Cr‐Cys_ with WMH volumes being stronger among those with high DII scores (*p* for interaction < 0.05). Regarding the DTI metrics, renal dysfunction was associated with decreased FA and increased MD in both DII groups, and the effects of eGFR_Cys_ and eGFR_Cr‐Cys_ on FA and eGFR_Cr‐Cys_ on MD were stronger in participants with high DII scores (*p* for interaction < 0.05) (Table S7).

Next, we examined the joint association of multidimensional renal function biomarkers and chronic comorbidities (hypertension, diabetes, and dyslipidaemia) with CSVD. Compared with the healthy group, renal dysfunction, as reflected by eGFR_Cr_, eGFR_Cys_, eGFR_Cr‐Cys_, and BUN levels, was associated with increased WMH volumes in both disease‐status strata. The positive associations of eGFR_Cys_, eGFR_Cr‐Cys_, eGFR_Cr_, and BUN with WMH volumes were stronger among those without hypertension, diabetes, or dyslipidaemia (Table S8). However, no modification effects of disease status were observed for the associations between multidimensional renal function biomarkers and FA or MD (Tables S9 and S10), indicating consistent associations across all subgroups.

## 4 Discussion

This large‐scale epidemiological study found that multidimensional renal dysfunction, as defined by eGFR_Cr_, eGFR_Cys_, eGFR_Cr‐Cys_, and BUN, was associated with increased risk of CSVD. In the whole cohort, renal dysfunction was associated with increased WMH volume and white matter MD, and decreased white matter FA. In the sub‐cohort, renal dysfunction was associated with increased risk of lacunes, WMHs, EPVS, and CMBs. MR analyses revealed significant associations between genetically determined eGFR and WMH volume, supporting a causal link between multidimensional renal dysfunction biomarkers and CSVD risk.

WMH volumes indicate pathological alterations observed on MRI and are commonly attributed to CSVD in adults [1]. The DTI measures, FA and MD, assess the microstructural integrity of the white matter and are expected to be more sensitive to interference with normal function and structure than measures of pathological alterations alone [7]. Higher white matter MD and lower FA values indicate abnormal white matter integrity [16]. Studies examining the association between renal function biomarkers and CSVD have mainly focused on patients with specific diseases and conventional CSVD MRI features. Xu et al. explored the association between eGFR and CSVD in patients with primary intracerebral hemorrhage and found that the eGFR value was adversely associated with the CSVD burden score [22]. Tomas Månsson et al. evaluated the association between CKD (assessed using the eGFR) and CSVD in those with hypertension and revealed that CKD was positively associated with cerebral microbleeds and cortical atrophy [23]. Hirao et al. found that serum cystatin C levels were associated with the volume of periventricular hyperintensity in patients with cognitive impairment [24]. In addition to the aforementioned evidence, our study provides new insights that multidimensional renal dysfunction biomarkers, as reflected by eGFR_Cr_, eGFR_Cys_, eGFR_Cr‐Cys_, and BUN, may be associated with emerging features (DTI metrics: white matter FA and MD), which may be considered as a more sensitive or subtle measure of white matter disease compared with macrostructural MRI markers. Identifying early declines in white matter integrity is critical for preventing the progression of brain pathologies before they become irreversible.

The underlying mechanisms linking renal dysfunction to CSVD risk remain unclear; however, growing evidence supports the existence of a bidirectional kidney–brain axis [34]. CKD is frequently accompanied by neurological disorders, suggesting shared or interacting pathophysiological pathways. Several mechanisms may explain the potential link between renal dysfunction and CSVD. Among them, hemodynamic alterations appear to play a key role. In patients with renal dysfunction, arterial stiffening and microvascular impairment have been shown to induce damage to the cerebral microcirculation, a pathological process correlated with the development of CSVD [35]. Moreover, CKD patients exhibit elevated levels of global cerebral blood flow, a hemodynamic alteration that may exert a profound impact on cerebral physiological function [20, 36]. Another mechanism linked to renal dysfunction and CSVD is inflammation. The kidney helps maintain immune system homeostasis by clearing circulating cytokines and bacterial antigens, thereby reducing inflammation and modulating immune cell activation [37, 38]. Animal studies have shown that CKD can lead to increased production of circulating pro‐inflammatory cytokines, such as IL‐1, IL‐6, IL‐18, IFN‐γ, and TNF‐α [39]. Additionally, epidemiological studies have demonstrated that circulating inflammatory biomarkers are increased in individuals with CKD and are independent risk factors for incident cardiovascular events and death [13, 40, 41, 42]. Thus, it is reasonable to hypothesize that renal dysfunction may alter CSVD progression.

Our findings demonstrate that renal dysfunction is associated with an increased risk of EPVS, suggesting an intriguing conceptual link between renal filtration failure and impaired cerebral clearance, as EPVS are increasingly regarded as markers of glymphatic dysfunction [43]. The glymphatic system, responsible for clearing central nervous system waste products, is most active during deep sleep [44]. Considering the endothelial dysfunction and sleep alterations in patients with renal dysfunction, it is plausible that glymphatic fluid clearance in the brain may be suppressed, thereby facilitating the accumulation of potentially neurotoxic waste products [45].

Our study explored the combined association of multidimensional renal function biomarkers and DII scores with the risk of CSVD. Studies have demonstrated that diets can exert either pro‐ or anti‐inflammatory effects [46, 47]. High intake of trans and saturated fatty acids is associated with elevated levels of inflammatory biomarkers, whereas high intake of fruits, vegetables, vitamin C, and vitamin E is associated with reduced levels of inflammatory biomarkers [48, 49]. Therefore, diet is a lifestyle factor that modulates inflammation. Our findings suggest that an anti‐inflammatory diet may help mitigate the adverse effects of inflammation caused by renal dysfunction on CSVD risk, indicating that dietary modification could serve as a potential preventive strategy for CSVD. Nevertheless, further research is still needed to investigate the mediating role of systemic inflammatory biomarkers in the association between renal dysfunction and CSVD MRI markers.

Our study had two notable strengths. First, we leveraged a large‐scale UK Biobank cohort from UK Biobank to accurately measure multidimensional renal function biomarkers and CSVD risk in each participant. Second, we extracted GWAS data, offering insights into the potential causal associations between renal dysfunction and CSVD. Our study has a few limitations. First, the UK Biobank is a volunteer cohort [50], and its participants are healthier than the general population, which may have resulted in an underestimation of the impact of renal dysfunction. Second, because of the substantial workload, CSVD MRI features were evaluated in 9786 participants rather than in the whole cohort by neuroradiologists. Nonetheless, the random sampling design and rigorous, blinded image evaluations by independent neuroradiologists helped to mitigate selection bias and ensure the robustness of our findings. Third, although we utilized the UK Biobank, one of the largest population‐based cohorts to explore the associations between renal dysfunction and CSVD, the lack of information on other ethnic backgrounds may have introduced selection bias. Further multicentre cohort studies involving participants with diverse demographic characteristics are still required to confirm our findings. Fourth, the current analysis focuses primarily on total WMH volume. However, it is well recognized that periventricular WMH and deep WMH often reflect distinct pathophysiological mechanisms; further study is still warranted to examine the association between renal function and WMH volume in different brain regions. Additionally, the use of dichotomous variables (Yes/No) for conditions such as hypertension and dyslipidemia constitutes a methodological limitation. Although this approach facilitates standardized and consistent reporting, it does not capture important clinical nuances such as disease duration and severity. Future studies incorporating graded measures of these factors are needed to clarify our findings. Finally, the impact of other renal function biomarkers, such as the urinary albumin‐to‐creatinine ratio, on CSVD risk warrants further investigation [51, 52].

## 5 Conclusions

This large‐scale epidemiological study, integrating two cohort studies from the UK Biobank, demonstrated that multidimensional renal dysfunction biomarkers are independently associated with CSVD, suggesting that renal dysfunction may contribute to CSVD pathogenesis in adults.

## Funding

This work was supported by Shanghai Science and Technology Program (23DZ2291900), Clinical Research Plan of SHDC (SHDC2022CRW003), the Innovative Research Team of High‐level Local Universities in Shanghai (SHSMU‐ZLCX20211602), National Natural Science Foundation of China (NSFC 82525023, W2411087, U22A20295, 82061130224, M‐0671), the Fundamental Research Funds for the Central Universities (YG2023QNA01), Shanghai Municipal Education Commission‐Gaofeng Clinical Medical Grant Support (20181805).

## Ethics Statement

The UK Biobank was approved by the North West Multi‐centre Research Ethics Committee (21/NW/0157).

## Conflicts of Interest

Peiying Li is an Academic Editor of CNS Neuroscience and Therapeutics and a co‐author of this article. To minimize bias, she was excluded from all editorial decision‐making related to the acceptance of this article for publication.

## Supporting information


**Data S1:** cns70802‐sup‐0001‐Supinfo.docx.

## Data Availability

The data that support the findings of this study are available on request from the corresponding author. The data are not publicly available due to privacy or ethical restrictions.
