# Supplementary material for: Association Between Renal Dysfunction and Cerebral Small Vessel Disease: A Prospective Cohort Study From the UK Biobank
Source: CNS Neurosci Ther. 2026 Feb 24;32(2):e70802. doi: 10.1002/cns.70802 (PMC12930275; doi:10.1002/cns.70802)
Supplement: Supplementary file 1 — Data S1: cns70802‐sup‐0001‐Supinfo.docx. [file CNS-32-e70802-s001.docx]

**SUPPLEMENTARY DOCUMENTS**

1. **Supplemental Appendices**

Appendix 1: Detailed measurement and calculation of the renal function

1. **Supplemental Figures**

Figure S1. Restricted cubic spline model of renal function and the first principal component of fractional anisotropy and mean diffusivity in the whole cohort

Figure S2. Restricted cubic spline model of renal function and CSVD burden in sub-cohort

Figure S3. Mendelian Randomization Estimates for the Association between Renal Function and cerebral small vessel disease marker

Figure S4. Scatter plots and forest plot results of renal function on the cerebral small vessel disease marker

1. **Supplemental Tables**

Table S1. Neurological Disorders Used as Exclusion Criteria

Table S2. Food patterns used in this study for calculating the dietary inflammatory index, and their respective inflammatory effect scores

Table S3. Distribution of renal function in participants

Table S4. Characteristics of the participants include and exclude the sub-cohort

Table S5 The association between renal function and CSVD further adjusting for sleep disorder and medication history

Table S6. Distribution of CSVD across strata of renal function status in sub-cohort

Table S7. The interaction of dietary inflammation index on the associations between renal function and CSVD markers in the whole cohort

Table S8. The association between renal function and white matter hyperintensity volumes among subgroups in the whole cohort

Table S9. The association between renal function and fractional anisotropy among subgroups in the whole cohort

Table S10. The association between renal function and mean diffusivity among subgroups in the whole cohort

**SUPPLEMENTAL APPENDICES**

**Appendix 1: Detailed measurement and calculation of the renal function**

The formula of renal function calculate was: eGFR = µ×min(Scr/κ, 1)a1×max(Scr/κ, 1)a2×min(Scr/0.8, 1)b1×max(Scr/0.8, 1)^b2^×c^Age^×d[if female]. Here, κ = 0.7 for women and 0.9 for men, µ=142, a1=-0.241F/-0.302M, a2=-1.2, c=0.9938, and d=1.012 when calculating eGFR_cr. µ=133, b1=-0.499, b2=-1.328, c=0.9962, and d=0.932 when calculating eGFR_cys. µ=135, a1=-0.219F/-0.144M, a2=-0.544, b1=-0.323, b2=-0.778, c=0.9961, and d=0.963 when calculating eGFR. BUN (mg/dl) was calculated by Urea (mmol/l) × 2.801.

**Supplemental Figures**


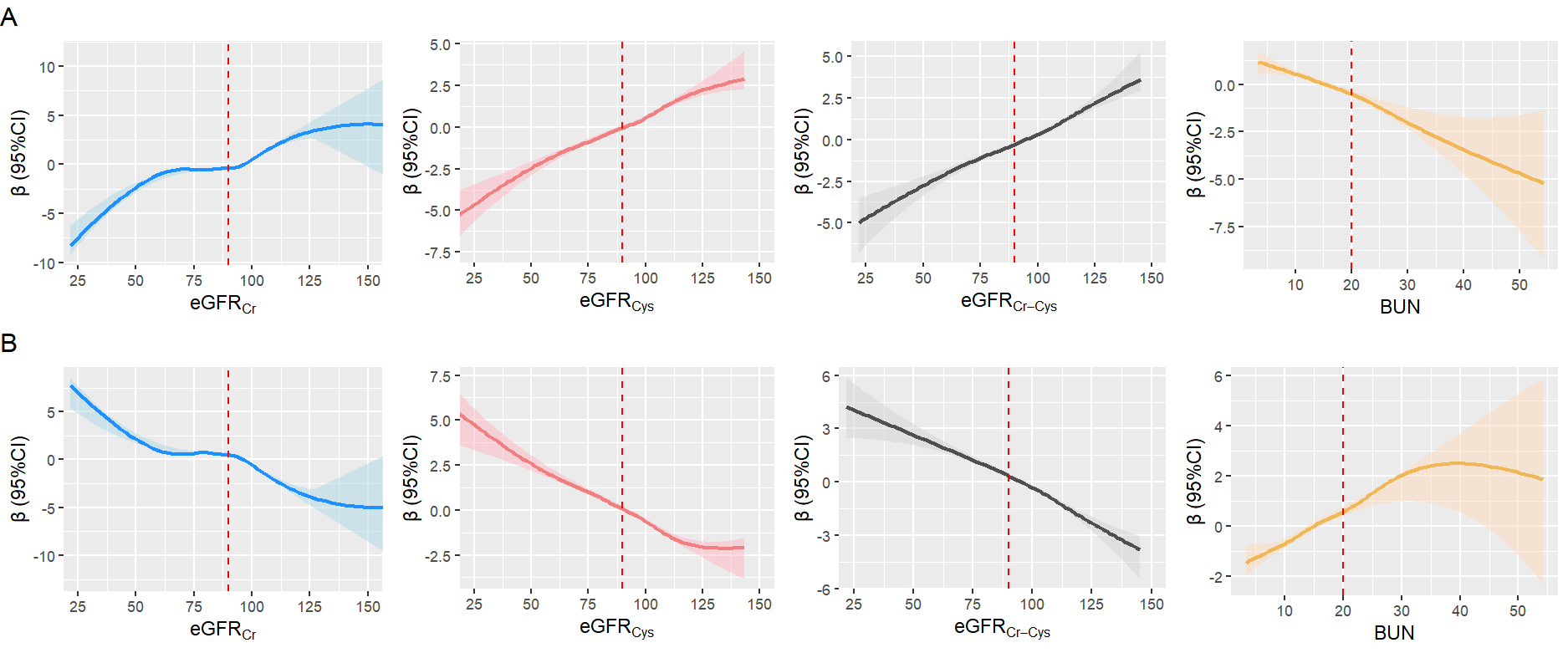


**Figure S1. Restricted cubic spline model of renal function and the first principal component of fractional anisotropy and mean diffusivity in the whole cohort**

Note: (A) Restricted cubic spline model of renal function exposure and the first principal component of FA in the whole cohort. (B) Restricted cubic C spline model of renal function exposure and the first principal component of MD in the whole cohort.

1) Abbreviations: eGFR_Cr_: estimated Glomerular Filtration Rate based on Creatinine; eGFR_Cys_: estimated Glomerular Filtration Rate based on Cystatin C; eGFR_Cr-Cys_: estimated Glomerular Filtration Rate based on Creatinine and Cystatin C; BUN: blood urea nitrogen; FA: Fractional anisotropy; MD: Mean diffusivity.

2) The blue line represents eGFR_Cr_, the red line represents eGFR_Cys_, the black line represents eGFR_Cr-Cys_, and the yellow line represents BUN. Dotted line represents health status, eGFR_Cr_ ≥ 90, eGFR_Cys_ ≥90, eGFR_Cr-Cys_ ≥90, and BUN < 20.

3) eGFR_Cr_, eGFR_Cys_, and eGFR_Cr-Cys_ models adjusted for household income, BMI, race, tobacco smoking, alcohol drinking, and brain volume; BUN model adjusted for age, sex, household income, BMI, race, tobacco smoking, alcohol drinking, and brain volume.


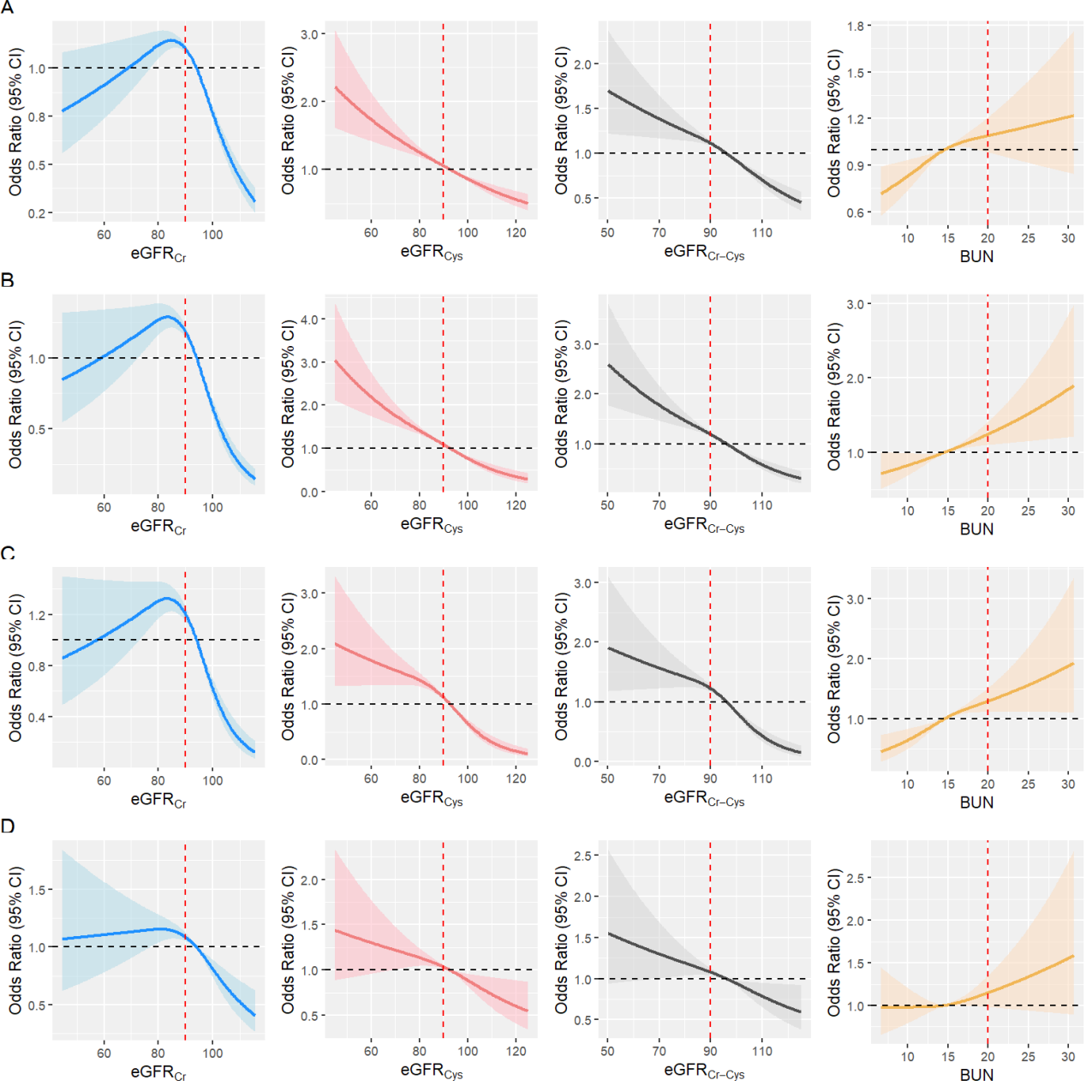


**Figure S2. Restricted cubic spline model of renal function and CSVD burden in sub-cohort**

Note: (A) Restricted cubic spline model of renal function and lacunes in sub-cohort. (B) Restricted cubic spline model of renal function and WMHs in sub-cohort. (C) Restricted cubic spline model of renal function and EPVS in sub-cohort. (D) Restricted cubic spline model of renal function and CMBs sub-cohort.

1) Abbreviations: eGFR_Cr_: estimated Glomerular Filtration Rate based on Creatinine; eGFR_Cys_: estimated Glomerular Filtration Rate based on Cystatin C; eGFR_Cr-Cys_: estimated Glomerular Filtration Rate based on Creatinine and Cystatin C; BUN: blood urea nitrogen; WMHs: White matter hyperintensities; EPVS: enlarged perivascular space; CMBs: cerebral microbleeds.

2) The blue line represents eGFR_Cr_, the red line represents eGFR_Cys_, the black line represents eGFR_Cr-Cys_, and the yellow line represents BUN. Dotted line represents health status, eGFR_Cr_ ≥ 90, eGFR_Cys_ ≥90, eGFR_Cr-Cys_ ≥90, and BUN < 20.

**Figure S3. Mendelian Randomization Estimates for the Association between Renal Function and cerebral small vessel disease marker**

Note: 1) Abbreviations: eGFR_Cr_: estimated Glomerular Filtration Rate based on Creatinine; eGFR_Cys_: estimated Glomerular Filtration Rate based on Cystatin C; WMH: White matter hyperintensities. IVW: inverse-variance weighted; WME: weighted median.

1. * There are statistically significant differences.


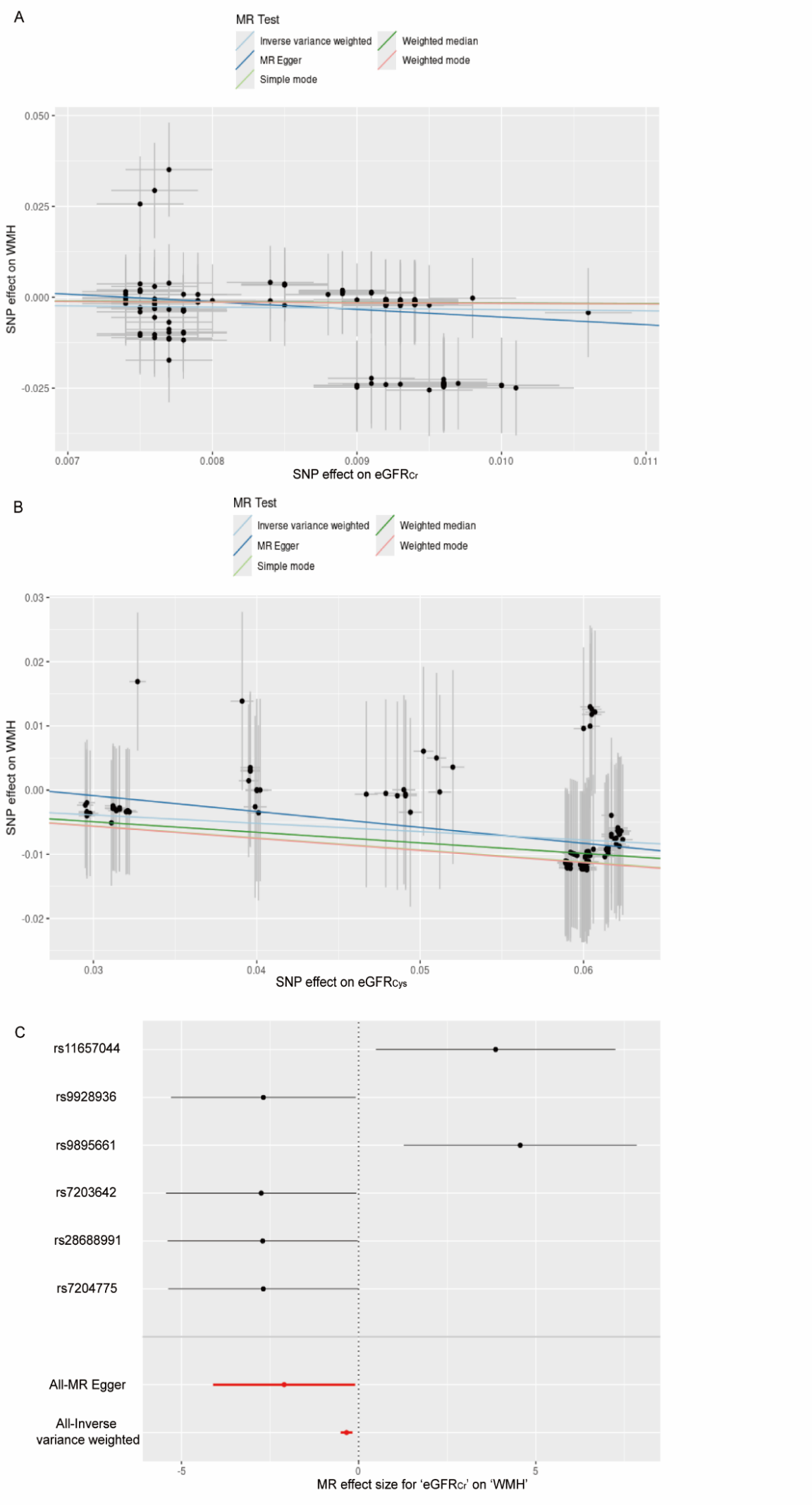


**Figure S4. Scatter plots and forest plot results of renal function on the cerebral small vessel disease marker**

Note: (A) Scatter plots of eGFR_Cr_ on the WMH volume. The slope of each line corresponding to the estimated MR effect in different models. (B) Scatter plots of eGFR_Cys_ on the WMH volume. The slope of each line corresponding to the estimated MR effect in different models. (C) Forest plots with the estimated MR effect of eGFR_Cr_ on the WMH volume in IVW models (*P*-value < 0.05).

**Supplemental Tables**

**Table S1. Neurological Disorders Used as Exclusion Criteria**

| **Neurological disorder** | **Self-reported codes (Field ID 20002) or ICD10 codes** |
| --- | --- |
| Parkinson’s disease | 1262 |
| Dementia or Alzheimer’s disease | 1263 |
| Chronic degenerative neurological | 1258 |
| Guillain-Barré syndrome | 1256 |
| Multiple Sclerosis | 1261 |
| Other demyelinating disease | 1397 |
| Brain cancer | 1032 |
| Brain/intracranial abscess | 1245 |
| Cerebral aneurysm | 1425 |
| Cerebral palsy | 1433 |
| Encephalitis | 1246 |
| Epilepsy | 1264 |
| Head injury | 1266 |
| Infections of the nervous system | 1244 |
| Meningeal cancer | 1031 |
| Meningioma (benign) | 1659 |
| Meningitis | 1247 |
| Motor Neuron Disease | 1259 |
| Neurological injury/trauma | 1240 |
| Spina bifida | 1524 |
| Subdural hematoma | 1083 |
| Subarachnoid haemorrhage | I60 |
| Intracerebral haemorrhage | I61 |
| Other nontraumatic intracranial haemorrhage | I62 |
| Cerebral infarction | I63 |
| Stroke, not specified as haemorrhage or infarction | I64 |
| Occlusion and stenosis of precerebral arteries, not resulting in cerebral infarction | I65 |
| Occlusion and stenosis of cerebral arteries, not resulting in cerebral infarction | I66 |
| Other cerebrovascular diseases | I67 |
| Cerebrovascular disorders in diseases classified elsewhere | I68 |
| Sequelae of cerebrovascular disease | I69 |

**Table S2. Food patterns used in this study for calculating the dietary inflammatory index, and their respective inflammatory effect scores**

| **Field ID** | **Items** | **Inflammatory effect score*** |
| --- | --- | --- |
| 26002 | Total energy (kcal) | 0.180 |
| 26005 | Protein(g) | 0.021 |
| 26008 | Total fat (g) | 0.298 |
| 26013 | Carbohydrate (g) | 0.097 |
| 26014 | Saturated fat (g) | 0.373 |
| 26015 | n-3 fatty acids (g) | -0.436 |
| 26016 | n-6 fatty acids (g) | -0.159 |
| 26017 | Fibre (g) | -0.663 |
| 26019 | Iron (mg) | 0.032 |
| 26020 | Vitamin B6(μg) | -0.365 |
| 26021 | Vitamin B12(μg) | 0.106 |
| 26022 | Folate (μg) | -0.190 |
| 26023 | Vitamin C(mg) | –0.424 |
| 26025 | Magnesium(mg) | –0.484 |
| 26027 | β-Carotene (μg) | -0.584 |
| 26028 | Vitamin E(mg) | –0.419 |
| 26029 | Vitamin D (ug) | -0.446 |
| 26030 | Alcohol (g) | -0.278 |
| 26032 | Monounsaturated fat (g) | -0.009 |
| 26033 | Zn (mg) | -0.313 |
| 26034 | Thiamin (mg) | -0.098 |
| 26035 | Riboflavin (mg) | -0.068 |
| 26037 | Cholesterol (mg) | 0.110 |
| 26054 | Niacin (mg) | -0.246 |
| 26058 | Selenium (μg) | -0.191 |
| 26061 | Vitamin A (RE) | -0.401 |
| 26141 | Tea (g) | –0.536 |
| 26155 | Trans fatty acid (g) | 0.229 |
| 100007 | Polysaturated fat (g) | –0.337 |

*Positive scores indicated that the corresponding food parameters were associated with a pro-inflammatory effect, while negative scores indicated that the corresponding food parameters were associated with anti-inflammatory effect.

**Table S3. Distribution of renal function in participants**

| **Renal function** | **N** | **Mean** | **SD** | **5th percentile** | **25th percentile** | **50th percentile** | **75th percentile** | **95th percentile** |
| --- | --- | --- | --- | --- | --- | --- | --- | --- |
| **The whole cohort** | | | | | | | | |
| **eGFR_Cr_** | 43314 | 91.75 | 11.46 | 70.31 | 84.75 | 93.95 | 99.92 | 107.14 |
| **eGFR_Cys_** | 43314 | 90.83 | 13.94 | 66.51 | 81.15 | 91.99 | 101.64 | 110.87 |
| **eGFR_Cr-Cys_** | 43314 | 95.12 | 12.41 | 73.38 | 87.07 | 96.10 | 104.30 | 113.29 |
| **BUN** | 43314 | 14.87 | 3.45 | 9.83 | 12.52 | 14.57 | 16.83 | 20.92 |
| **Sub-cohort** | | | | | | | | |
| **eGFR_Cr_** | 9786 | 91.85 | 11.34 | 70.65 | 84.84 | 94.07 | 99.96 | 107.06 |
| **eGFR_Cys_** | 9786 | 91.33 | 13.62 | 67.42 | 81.81 | 92.73 | 101.98 | 110.79 |
| **eGFR_Cr-Cys_** | 9786 | 95.44 | 12.15 | 74.15 | 87.55 | 96.29 | 104.51 | 113.11 |
| **BUN** | 9786 | 14.83 | 3.40 | 9.80 | 12.52 | 14.57 | 16.75 | 20.81 |

Note: Abbreviations: eGFR_Cr_: estimated Glomerular Filtration Rate based on Creatinine; eGFR_Cys_: estimated Glomerular Filtration Rate based on Cystatin C; eGFR_Cr-Cys_: estimated Glomerular Filtration Rate based on Creatinine and Cystatin C; BUN: blood urea nitrogen; SD: standard deviation

**Table S4. Characteristics of the participants include and exclude the sub-cohort**

| **Characteristics** | **Sub-cohort** | **Exclude cohort** |
| --- | --- | --- |
|  | **Mean±SD or N (%)** | |
| **Age** | 54.70(7.48) | 55.07(7.55) |
| **Sex** |  |  |
| Male | 4626(47.27) | 15760(47.01) |
| Female | 5160(52.73) | 17768(52.99) |
| **BMI, kg/m^2^** | 26.40(4.10) | 26.61(4.25) |
| **Race** |  |  |
| White British | 8937(91.34) | 30503(91.06) |
| Other | 847(8.66) | 2996(8.94) |
| **Tobacco smoking** |  |  |
| Never smoked | 6189(63.26) | 20162(60.15) |
| Previous | 3128(31.97) | 11094(33.10) |
| Current | 448(4.58) | 2200(6.56) |
| Unknown | 19(0.19) | 63(0.19) |
| **Alcohol drinking** |  |  |
| Never drinked | 245(2.50) | 805(2.40) |
| Previous | 154(1.57) | 737(2.20) |
| Current | 9383(95.90) | 31966(95.37) |
| Unknown | 2(0.02) | 11(0.03) |
| **Household income range, £** |  |  |
| <18000 | 891(9.24) | 3602(10.90) |
| 18000~30999 | 1925(19.96) | 6666(20.17) |
| 31000~51999 | 2687(27.86) | 9113(27.57) |
| 52000~100000 | 2651(27.49) | 8476(25.64) |
| >100000 | 713(7.39) | 2343(7.09) |
| Unknown | 776(8.05) | 2857(8.64) |
| **Hypertension** |  |  |
| Yes | 1791(21.78) | 7695(26.52) |
| No | 6433(78.22) | 21322(73.48) |
| **Diabetes** |  |  |
| Yes | 345(4.20) | 1669(5.75) |
| No | 7879(95.80) | 27348(94.25) |
| **Dyslipidemia** |  |  |
| Yes | 868(10.55) | 4052(13.96) |
| No | 7356(89.45) | 24965(86.04) |
| **Renal function** |  |  |
| eGFR_Cr_ | 91.85(11.34) | 91.72(11.49) |
| eGFR_Cys_ | 91.33(13.62) | 90.69(14.03) |
| eGFR_Cr-Cys_ | 95.44(12.15) | 95.03(12.49) |
| BUN | 14.83(3.40) | 14.88(3.46) |

Abbreviations BMI: Body Mass Index; eGFR_Cr_: estimated Glomerular Filtration Rate based on Creatinine; eGFR_Cys_: estimated Glomerular Filtration Rate based on Cystatin C; eGFR_Cr-Cys_: estimated Glomerular Filtration Rate based on Creatinine and Cystatin C; BUN: blood urea nitrogen.

**Table S5 The association between renal function and CSVD further adjusting for sleep disorder and medication history**

| **Renal function** | **All** | | |  | **Male** | | |  | **Female** | | |
| --- | --- | --- | --- | --- | --- | --- | --- | --- | --- | --- | --- |
|  | **β** | **95%CI** | ***P***  ***(P for FDR)*** |  | **β** | **95%CI** | ***P***  ***(P for FDR)*** |  | **β** | **95%CI** | ***P***  ***(P for FDR)*** |
| **WMH** | | | | | | | | | | | |
| **eGFR_Cr_^a^** | ref | | |  | ref | | |  | ref | | |
|  | 0.155 | 0.136, 0.175 | <0.001 |  | 0.166 | 0.138, 0.195 | <0.001 |  | 0.141 | 0.115, 0.167 | <0.001 |
| **eGFR_Cys_^a^** | ref | | |  | ref | | |  | ref | | |
|  | 0.309 | 0.289, 0.328 | <0.001 |  | 0.253 | 0.226, 0.281 | <0.001 |  | 0.340 | 0.314, 0.367 | <0.001 |
| **eGFR_Cr-Cys_^a^** | ref | | |  | ref | | |  | ref | | |
|  | 0.260 | 0.239, 0.280 | <0.001 |  | 0.228 | 0.199, 0.258 | <0.001 |  | 0.280 | 0.251, 0.308 | <0.001 |
| **BUN^b^** | ref | | |  | ref | | |  | ref | | |
|  | 0.008 | -0.026, 0.041 | 0.686 |  | -0.031 | -0.075, 0.013 | 0.259 |  | 0.067 | 0.014, 0.119 | 0.021 |
| **FA** | | | | | | | | | | | |
| **eGFR_Cr_^a^** | ref | | |  | ref | | |  | ref | | |
|  | -0.446 | -0.533, -0.359 | <0.001 |  | -0.481 | -0.610, -0.353 | <0.001 |  | -0.421 | -0.539, -0.304 | <0.001 |
| **eGFR_Cys_^a^** | ref | | |  | ref | | |  | ref | | |
|  | -0.773 | -0.860, -0.686 | <0.001 |  | -0.707 | -0.834, -0.580 | <0.001 |  | -0.872 | -0.992, -0.751 | <0.001 |
| **eGFR_Cr-Cys_^a^** | ref | | |  | ref | | |  | ref | | |
|  | -0.728 | -0.820, -0.637 | <0.001 |  | -0.684 | -0.816, -0.551 | <0.001 |  | -0.783 | -0.909, -0.656 | <0.001 |
| **BUN^b^** | ref | | |  | ref | | |  | ref | | |
|  | -0.273 | -0.430, -0.115 | 0.001 |  | -0.232 | -0.437, -0.259 | 0.043 |  | -0.332 | -0.579, -0.084 | 0.016 |
| **MD** | | | | | | | | | | | |
| **eGFR_Cr_^a^** | ref | | |  | ref | | |  | ref | | |
|  | 0.576 | 0.487, 0.664 | <0.001 |  | 0.633 | 0.501, 0.764 | <0.001 |  | 0.506 | 0.386, 0.625 | <0.001 |
| **eGFR_Cys_^a^** | ref | | |  | ref | | |  | ref | | |
|  | 1.124 | 1.035, 1.213 | <0.001 |  | 0.996 | 0.867, 1.125 | <0.001 |  | 1.132 | 1.010, 1.254 | <0.001 |
| **eGFR_Cr-Cys_^a^** | ref | | |  | ref | | |  | ref | | |
|  | 1.010 | 0.917, 1.104 | <0.001 |  | 0.971 | 0.835, 1.106 | <0.001 |  | 0.995 | 0.866, 1.124 | <0.001 |
| **BUN^b^** | ref | | |  | ref | | |  | ref | | |
|  | 0.094 | -0.063, 0.252 | 0.301 |  | 0.055 | -0.150, 0.261 | 0.669 |  | 0.157 | -0.092, 0.405 | 0.274 |

Note: 1) Abbreviations: eGFR_Cr_: estimated Glomerular Filtration Rate based on Creatinine; eGFR_Cys_: estimated Glomerular Filtration Rate based on Cystatin C; eGFR_Cr-Cys_: estimated Glomerular Filtration Rate based on Creatinine and Cystatin C; BUN: blood urea nitrogen; WMH: White matter hyperintensities.

2) ^a^ Models adjusted for household income, BMI, race, tobacco smoking, alcohol drinking, brain volume, hypertension, and sleep disorder.

^b^Models adjusted for age, sex, household income, BMI, race, tobacco smoking, alcohol drinking, brain volume, hypertension, and sleep disorder.

3) Defined by renal function: ref: eGFR_Cr_ ≥ 90, eGFR_Cys_ ≥90, eGFR_Cr-Cys_ ≥90, and BUN < 20.

**Table S6 Distribution of CSVD across strata of renal function status in sub-cohort**

| **CSVD, No./total No.(%)** | **Renal function variable** | | | | | | | |
| --- | --- | --- | --- | --- | --- | --- | --- | --- |
|  | **eGFR_Cr_ group** | | **eGFR_Cys_ group** | | **eGFR_Cr-Cys_ group** | | **BUN group** | |
|  | Healthy | Renal dysfunction | Healthy | Renal dysfunction | Healthy | Renal dysfunction | Healthy | Renal dysfunction |
| **lacunes** | | | | | | | | |
| **Yes** | 3749/5761(65.1) | 2369/3366(70.4) | 3216/5138(62.6) | 2908/3996(72.8) | 3999/6266(63.8) | 2115/2856(74.1) | 5660/8475(66.8) | 458/651(70.4) |
| **No** | 2012/5761(34.9) | 997/3366(29.6) | 1922/5138(37.4) | 1088/3996(27.2) | 2267/6266(36.2) | 741/2856(25.9) | 2815/8475(33.2) | 193/651(29.6) |
| **WMHs** | | | | | | | | |
| **Yes** | 609/5761(10.6) | 505/3366(15.0) | 465/5138(9.1) | 650/3996(16.3) | 614/6266(9.8) | 499/2856(17.5) | 1006/8475(11.9) | 108/651(16.6) |
| **No** | 5152/5761(89.4) | 2861/3366(85.0) | 4673/5138(90.9) | 3346/3996(83.7) | 5652/6266(90.2) | 2357/2856(82.5) | 7469/8475(88.1) | 543/651(83.4) |
| **EPVS** | | | | | | | | |
| **Yes** | 330/5761(5.7) | 305/3366(9.1) | 236/5138(4.6) | 398/3996(10.0) | 338/6266(5.4) | 295/2856(10.3) | 571/8475(6.7) | 64/651(9.8) |
| **No** | 5431/5761(94.3) | 3061/3366(90.9) | 4902/5138(95.4) | 3598/3996(90.0) | 5928/6266(94.6) | 2561/2856(89.7) | 7904/8475(93.3) | 587/651(90.2) |
| **CMBs** | | | | | | | | |
| **Yes** | 403/5761(7.0) | 281/3366(8.3) | 344/5138(6.7) | 342/3996(8.6) | 431/6266(6.9) | 253/2856(8.9) | 619/8475(7.3) | 65/651(10.0) |
| **No** | 5358/5761(93.0) | 3085/3366(91.7) | 4794/5138(93.3) | 3654/3996(91.4) | 5835/6266(93.1) | 2603/2856(91.1) | 7856/8475(92.7) | 586/651(90.0) |
| **CSVD burden score** | | | | | | | | |
| **>1** | 1004/5761(17.4) | 804/3366(23.9) | 769/5138(15.0) | 1039/3996(26.0) | 1015/6266(16.2) | 790/2856(27.7) | 1636/8475 (19.3) | 172/651(26.4) |
| **≤1** | 4757/5761(82.6) | 2562/3366(76.1) | 4369/5138(85.0) | 2957/3996(74.0) | 5251/6266(83.8) | 2066/2856(72.3) | 6829/8475(80.7) | 479/651(73.6) |

Note: Abbreviations: eGFR_Cr_: estimated Glomerular Filtration Rate based on Creatinine; eGFR_Cys_: estimated Glomerular Filtration Rate based on Cystatin C; eGFR_Cr-Cys_: estimated Glomerular Filtration Rate based on Creatinine and Cystatin C; BUN: blood urea nitrogen; CSVD: cerebral small vessel disease; WMHs: white matter hyperintensities; EPVS: enlarged perivascular space; CMBs: cerebral microbleeds.

**Table S7 The interaction of dietary inflammation index on the associations between renal function and CSVD markers in the whole cohort**

| **Renal**  **function** | **WMH** | | | |  | **FA** | | | |  | **MD** | | | |
| --- | --- | --- | --- | --- | --- | --- | --- | --- | --- | --- | --- | --- | --- | --- |
|  | **DII** | **β** | **95%CI** | ***P* for**  **interaction** |  | **DII** | **β** | **95%CI** | ***P* for**  **interaction** |  | **DII** | **β** | **95%CI** | ***P* for**  **interaction** |
| **eGFR_Cr_ ^a^** | high | ref | | 0.046 |  | high | ref | | 0.115 |  | high | ref | | 0.276 |
|  |  | 0.222 | 0.177, 03268 |  |  |  | -0.677 | -0.879, -0.475 |  |  |  | 0.782 | 0.574, 0.989 |  |
|  | low | ref | |  |  | low | ref | |  |  | low | ref | |  |
|  |  | 0.165 | 0.136, 0.195 |  |  |  | -0.490 | -0.621, -0.360 |  |  |  | 0.653 | 0.519, 0.787 |  |
| **eGFR_Cys_ ^a^** | high | ref | | 0.207 |  | high | ref | | 0.049 |  | high | ref | | 0.117 |
|  |  | 0.383 | 0.338, 0.428 |  |  |  | -1.102 | -1.303, -0.901 |  |  |  | 1.454 | 1.248, 1.660 |  |
|  | low | ref | |  |  | low | ref | |  |  | low | ref | |  |
|  |  | 0.348 | 0.319, 0.377 |  |  |  | -0.907 | -1.037, -0.776 |  |  |  | 1.303 | 1.170, 1.436 |  |
| **eGFR_Cr-Cys_ ^a^** | high | ref | | 0.038 |  | high | ref | | 0.043 |  | high | ref | | 0.040 |
|  |  | 0.347 | 0.300, 0.395 |  |  |  | -1.026 | -1.238, -0.814 |  |  |  | 1.363 | 1.146, 1.580 |  |
|  | low | ref | |  |  | low | ref | |  |  | low | ref | |  |
|  |  | 0.285 | 0.254, 0.316 |  |  |  | -0.804 | -0.942, -0.665 |  |  |  | 1.128 | 0.986, 1.269 |  |
| **BUN ^b^** | high | ref | | 0.111 |  | high | ref | | 0.304 |  | high | ref | | 0.646 |
|  |  | 0.086 | 0.007, 0.164 |  |  |  | -0.161 | -0.530, 0.209 |  |  |  | 0.280 | -0.089, 0.649 |  |
|  | low | ref | |  |  | low | ref | |  |  | low | ref | |  |
|  |  | 0.015 | -0.036, 0.070 |  |  |  | -0.456 | -0.698, -0.213 |  |  |  | 0.236 | 0.001, 0.487 |  |

Note: 1) Abbreviations: eGFR_Cr_: estimated Glomerular Filtration Rate based on Creatinine; eGFR_Cys_: estimated Glomerular Filtration Rate based on Cystatin C; eGFR_Cr-Cys_: estimated Glomerular Filtration Rate based on Creatinine and Cystatin C; BUN: blood urea nitrogen; WMH: White matter hyperintensity; FA: Fractional anisotropy; MD: Mean diffusivity; CSVD: Cerebral Small Vascular Disease; DII: dietary inflammation index.

2) eGFR_Cr_, eGFR_Cys_, and eGFR_Cr-Cys_ models adjusted for household income, BMI, race, tobacco smoking, alcohol drinking, and brain volume.

BUN models adjusted for age, sex, household income, BMI, race, tobacco smoking, alcohol drinking, and brain volume.

3) The population was classified using a cutoff value of 0 for the Dietary Inflammatory Index. Groups were defined based on renal function.

**Table S8 The association between renal function and white matter hyperintensity volumes among subgroups in the whole cohort**

| **Renal**  **function** | **Hypertension** | | | | |  | **Diabetes** | | | | |  | **Dyslipidemia** | | | | |
| --- | --- | --- | --- | --- | --- | --- | --- | --- | --- | --- | --- | --- | --- | --- | --- | --- | --- |
|  | **Group** | **β** | **95%CI** | | ***P* for**  **interaction** |  | **Group** | **β** | **95%CI** | | ***P* for**  **interaction** |  | **Group** | **β** | **95%CI** | | ***P* for**  **interaction** |
| **eGFR_Cr_ ^a^** | Yes | ref |  |  | 0.514 |  | Yes | ref |  |  | 0.164 |  | Yes | ref |  |  | 0.007 |
|  |  | 0.152 | 0.111, 0.193 | |  |  |  | 0.109 | 0.180, 0.200 | |  |  |  | 0.104 | 0.048, 0.160 | |  |
|  | No | ref |  |  |  |  | No | ref |  |  |  |  | No | ref |  |  |  |
|  |  | 0.155 | 0.133, 0.177 | |  |  |  | 0.181 | 0.160, 0.201 | |  |  |  | 0.174 | 0.153, 0.195 | |  |
| **eGFR_Cys_ ^a^** | Yes | ref |  |  | <0.001 |  | Yes | ref |  |  | <0.001 |  | Yes | ref | | | <0.001 |
|  |  | 0.228 | 0.185, 0.270 | |  |  |  | 0.191 | 0.100, 0.283 | |  |  |  | 0.191 | 0.133, 0.249 | |  |
|  | No | ref |  |  |  |  | No | ref |  |  |  |  | No | ref | | |  |
|  |  | 0.333 | 0.311, 0.355 | |  |  |  | 0.353 | 0.333, 0.373 | |  |  |  | 0.351 | 0.330, 0.372 | |  |
| **eGFR_Cr-Cys_ ^a^** | Yes | ref |  |  | <0.001 |  | Yes | ref |  |  | 0.055 |  | Yes | ref | | | <0.001 |
|  |  | 0.201 | 0.160, 0.242 | |  |  |  | 0.227 | 0.137, 0.317 | |  |  |  | 0.189 | 0.133, 0.246 | |  |
|  | No | ref |  |  |  |  | No | ref |  |  |  |  | No | ref | | |  |
|  |  | 0.282 | 0.258, 0.305 | |  |  |  | 0.300 | 0.279, 0.321 | |  |  |  | 0.297 | 0.274, 0.319 | |  |
| **BUN ^b^** | Yes | ref |  |  | 0.810 |  | Yes | ref |  |  | 0.507 |  | Yes | ref | | | 0.034 |
|  |  | 0.022 | -0.040, 0.084 | |  |  |  | 0.007 | -0.125, 0.138 | |  |  |  | -0.037 | -0.120, 0.047 | |  |
|  | No | ref |  |  |  |  | No | ref |  |  |  |  | No | ref | | |  |
|  |  | 0.010 | -0.030, 0.050 | |  |  |  | 0.031 | -0.004, 0.066 | |  |  |  | 0.038 | 0.001, 0.075 | |  |

Note: 1) Abbreviations: eGFR_Cr_: estimated Glomerular Filtration Rate based on Creatinine; eGFR_Cys_: estimated Glomerular Filtration Rate based on Cystatin C; eGFR_Cr-Cys_: estimated Glomerular Filtration Rate based on Creatinine and Cystatin C; BUN: blood urea nitrogen.

2) ^a^ Models adjusted for household income, BMI, race, tobacco smoking, alcohol drinking, and brain volume.

^b^ Models adjusted for age, sex, household income, BMI, race, tobacco smoking, alcohol drinking, and brain volume.

1. Defined by renal function: ref: eGFR_Cr_ ≥ 90, eGFR_Cys_ ≥90, eGFR_Cr-Cys_ ≥90, and BUN < 20.

**Table S9 The association between renal function and fractional anisotropy among subgroups in the whole cohort**

| **Renal**  **function** | **Hypertension** | | | |  | **Diabetes** | | | |  | **Dyslipidemia** | | | |
| --- | --- | --- | --- | --- | --- | --- | --- | --- | --- | --- | --- | --- | --- | --- |
|  | **Group** | **β** | **95%CI** | ***P* for**  **interaction** |  | **Group** | **β** | **95%CI** | ***P* for**  **interaction** |  | **Group** | **β** | **95%CI** | ***P* for**  **interaction** |
| **eGFR_Cr_ ^a^** | Yes | ref | | 0.427 |  | Yes | ref | | 0.057 |  | Yes | ref | | 0.613 |
|  |  | -0.520 | -0.723, -0.318 |  |  |  | -0.154 | -0.615, 0.307 |  |  |  | -0.447 | -0.726, -0.169 |  |
|  | No | ref | |  |  | No | ref | |  |  | No | ref | |  |
|  |  | -0.420 | -0.516, -0.325 |  |  |  | -0.551 | -0.640, -0.461 |  |  |  | -0.498 | -0.590, -0.406 |  |
| **eGFR_Cys_ ^a^** | Yes | ref | | 0.871 |  | Yes | ref | | 0.350 |  | Yes | ref | | 0.204 |
|  |  | -0.814 | -1.022, -0.606 |  |  |  | -0.792 | -1.254, -0.330 |  |  |  | -0.739 | -1.024, -0.453 |  |
|  | No | ref | |  |  | No | ref | |  |  | No | ref | |  |
|  |  | -0.764 | -0.859, -0.669 |  |  |  | -0.916 | -1.005, -0.826 |  |  |  | -0.891 | -0.983, -0.799 |  |
| **eGFR_Cr-Cys_ ^a^** | Yes | ref | | 0.320 |  | Yes | ref | | 0.315 |  | Yes | ref | | 0.653 |
|  |  | -0.698 | -0.903, -0.494 |  |  |  | -0.725 | -1.179, -0.270 |  |  |  | -0.803 | -1.083, -0.522 |  |
|  | No | ref | |  |  | No | ref | |  |  | No | ref | |  |
|  |  | -0.743 | -0.845, -0.640 |  |  |  | -0.873 | -0.968, -0.779 |  |  |  | -0.826 | -0.923, -0.728 |  |
| **BUN ^b^** | Yes | ref | | 0.171 |  | Yes | ref | | 0.583 |  | Yes | ref | | 0.363 |
|  |  | -0.451 | -0.765, -0.138 |  |  |  | -0.220 | -0.909, 0.469 |  |  |  | -0.200 | -0.625, 0.226 |  |
|  | No | ref | |  |  | No | ref | |  |  | No | ref | |  |
|  |  | -0.218 | -0.402, -0.034 |  |  |  | -0.374 | -0.537, -0.211 |  |  |  | -0.383 | -0.555, -0.211 |  |

Note: 1) Abbreviations: eGFR_Cr_: estimated Glomerular Filtration Rate based on Creatinine; eGFR_Cys_: estimated Glomerular Filtration Rate based on Cystatin C; eGFR_Cr-Cys_: estimated Glomerular Filtration Rate based on Creatinine and Cystatin C; BUN: blood urea nitrogen.

2) ^a^ Models adjusted for household income, BMI, race, tobacco smoking, alcohol drinking, and brain volume.

^b^ Models adjusted for age, sex, household income, BMI, race, tobacco smoking, alcohol drinking, and brain volume.

3) Defined by renal function: ref: eGFR_Cr_ ≥ 90, eGFR_Cys_ ≥90, eGFR_Cr-Cys_ ≥90, and BUN < 20.

**Table S10 The association between renal function and mean diffusivity among subgroups in the whole cohort**

| **Renal**  **function** | **Hypertension** | | | |  | **Diabetes** | | | |  | **Dyslipidemia** | | | |
| --- | --- | --- | --- | --- | --- | --- | --- | --- | --- | --- | --- | --- | --- | --- |
|  | **Group** | **β** | **95%CI** | ***P* for**  **interaction** |  | **Group** | **β** | **95%CI** | ***P* for**  **interaction** |  | **Group** | **β** | **95%CI** | ***P* for**  **interaction** |
| **eGFR_Cr_ ^a^** | Yes | ref | | 0.009 |  | Yes | ref | | 0.409 |  | Yes | ref | | 0.366 |
|  |  | 0.803 | 0.587, 1.018 |  |  |  | 0.510 | 0.014, 1.006 |  |  |  | 0.765 | 0.468, 1.062 |  |
|  | No | ref | |  |  | No | ref | |  |  | No | ref | |  |
|  |  | 0.500 | 0.403, 0.596 |  |  |  | 0.683 | 0.591, 0.775 |  |  |  | 0.605 | 0.511, 0.700 |  |
| **eGFR_Cys_ ^a^** | Yes | ref | | 0.931 |  | Yes | ref | | 0.596 |  | Yes | ref | | 0.654 |
|  |  | 1.188 | 0.967, 1.409 |  |  |  | 1.264 | 0.769, 1.759 |  |  |  | 1.217 | 0.913, 1.522 |  |
|  | No | ref | |  |  | No | ref | |  |  | No | ref | |  |
|  |  | 1.103 | 1.008, 1.199 |  |  |  | 1.286 | 1.195, 1.377 |  |  |  | 1.237 | 1.143, 1.330 |  |
| **eGFR_Cr-Cys_ ^a^** | Yes | ref | | 0.964 |  | Yes | ref | | 0.382 |  | Yes | ref | | 0.539 |
|  |  | 1.063 | 0.846, 1.280 |  |  |  | 1.068 | 0.579, 1.557 |  |  |  | 1.244 | 0.945, 1.543 |  |
|  | No | ref | |  |  | No | ref | |  |  | No | ref | |  |
|  |  | 0.991 | 0.889, 1.094 |  |  |  | 1.177 | 1.080, 1.274 |  |  |  | 1.094 | 0.994, 1.194 |  |
| **BUN ^b^** | Yes | ref | | 0.153 |  | Yes | ref | | 0.826 |  | Yes | ref | | 0.745 |
|  |  | 0.247 | -0.082, 0.575 |  |  |  | 0.201 | -0.522, 0.924 |  |  |  | 0.100 | -0.345, 0.546 |  |
|  | No | ref | |  |  | No | ref | |  |  | No | ref | |  |
|  |  | 0.041 | -0.140, 0.221 |  |  |  | 0.189 | 0.026, 0.352 |  |  |  | 0.187 | 0.016, 0.358 |  |

Note: 1) Abbreviations: eGFR_Cr_: estimated Glomerular Filtration Rate based on Creatinine; eGFR_Cys_: estimated Glomerular Filtration Rate based on Cystatin C; eGFR_Cr-Cys_: estimated Glomerular Filtration Rate based on Creatinine and Cystatin C; BUN: blood urea nitrogen.

2) ^a^ Models adjusted for household income, BMI, race, tobacco smoking, alcohol drinking, and brain volume.

^b^ Models adjusted for age, sex, household income, BMI, race, tobacco smoking, alcohol drinking, and brain volume.

3) Defined by renal function: ref: eGFR_Cr_ ≥ 90, eGFR_Cys_ ≥90, eGFR_Cr-Cys_ ≥90, and BUN < 20.
